# Successful Treatment of Recurrent Hematometra With Ethanol Sclerotherapy: A Case Report

**DOI:** 10.1002/ccr3.72781

**Published:** 2026-05-20

**Authors:** Kojiro Tanabe, Fusako Komaru, Hirotaka Hamada, Hitoshi Niikura

**Affiliations:** ^1^ Department of Obstetrics and Gynecology Sendai Medical Center Sendai Japan; ^2^ Department of Obstetrics and Gynecology Tohoku University Hospital Sendai Japan

**Keywords:** ethanol sclerotherapy, minimal invasive, recurrent hematometra, repeated puncture

## Abstract

To our knowledge, this case report is the first to show that ethanol sclerotherapy can safely and effectively resolve recurrent hematometra caused by postradiotherapy stenosis and recurrent cervical cancer. For patients with poor overall condition in whom cervical drainage is not feasible, this minimally invasive approach may serve as a practical therapeutic option.

## 1 Introduction

Hematometra is a condition wherein cervical obstruction causes the accumulation of menstrual or uterine bleeding within the uterine cavity [1]. Reported causes include congenital cervical stenosis, postoperative adhesions after procedures such as conization or cesarean section, infection, and uterine malignancy. Although postmenopausal hematometra is often asymptomatic, patients may experience lower abdominal distention and pain from elevated intrauterine pressure. Uterine rupture secondary to hematometra has been documented, and on occasion may be fatal [2, 3], underscoring the need for timely intervention when symptoms arise or a markedly enlarged intrauterine cavity is detected [1].

Ethanol sclerotherapy has been used for various cystic or vascular lesions in other organs; however, its application in hematometra has not been described. We encountered a patient with recurrent hematometra caused by postradiotherapy cervical stenosis and recurrent cervical cancer. Cervical dilatation was impossible due to severe fibrotic obstruction, and the patient's poor overall condition made invasive procedures unsuitable. Although repeated transvaginal puncture provided temporary relief, the hematometra recurred; therefore, ethanol sclerotherapy was considered as a minimally invasive method to achieve a definitive resolution. To the best of our knowledge, this is the first report of the successful treatment of recurrent hematometra with ethanol sclerotherapy following radiotherapy for cervical cancer. Even in patients receiving best supportive care, this technique may enhance quality of life by reducing the need for repeated puncture and associated discomfort.

## 2 Case History and Examination

The patient was a multiparous woman in her early 80s with a history of myelodysplastic syndrome (MDS), diagnosed in her late 60s, requiring regular red blood cell and platelet transfusions. At the age of 78 years, she presented with abnormal genital bleeding and was diagnosed with stage IIB cervical cancer (FIGO 2018). Histopathology showed nonkeratinizing squamous cell carcinoma, and she underwent pelvic external beam radiotherapy (50 Gy), followed by intracavitary brachytherapy (18 Gy). She subsequently underwent regular clinical, cytological, and imaging follow‐up, with no evidence of recurrence.

**FIGURE 1:**
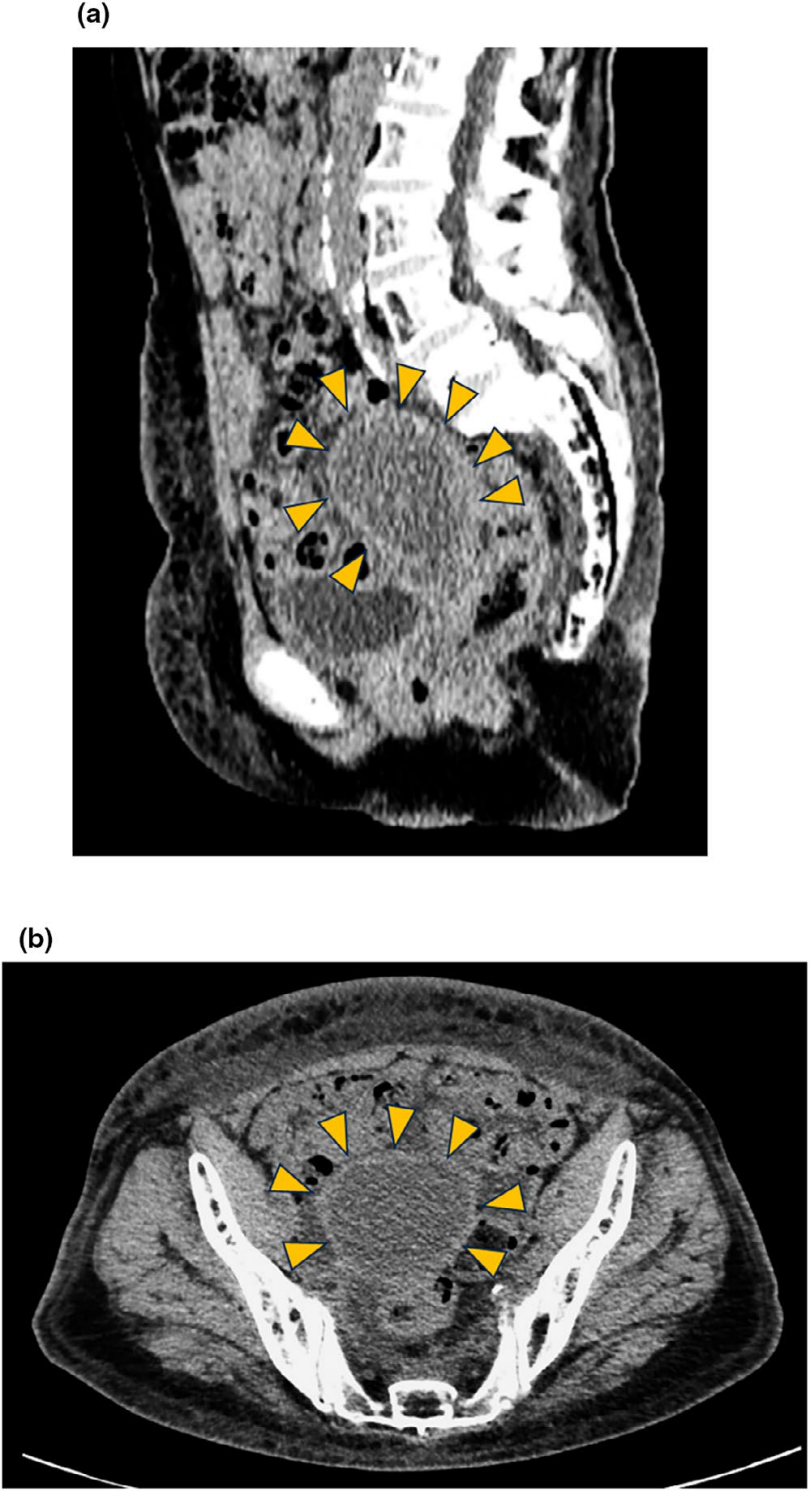
Computed tomography before ethanol sclerotherapy (a) Sagittal section (b) Axial section A large amount of fluid filled the uterine cavity, and the myometrium was thinned. Yellow arrowheads: Outline of the uterine body.

**FIGURE 2:**
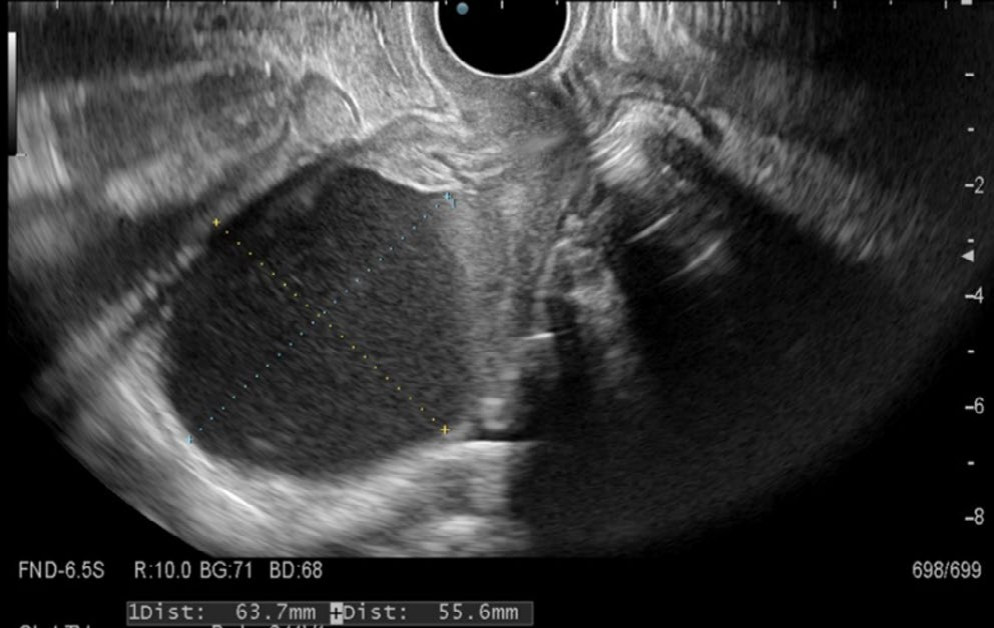
Transvaginal ultrasonography before ethanol sclerotherapy. Uterine cavity dilatation measuring 63 × 56 mm with a hypoechoic fluid collection and diffuse myometrial thinning is shown.

At the age of 81 years, she developed dull lower abdominal pain. Computed tomography demonstrated no abnormalities in the gastrointestinal and urinary systems; marked uterine cavity dilatation with myometrial thinning (Figure 1) was observed, as well as a new 1‐cm right lung nodule. The cervix was rigid, and the cervical canal was completely obstructed. Compared with a prior cervical diameter of 25 mm, transvaginal ultrasonography showed enlargement to 30 mm with hematometra. It also demonstrated uterine cavity dilatation to 63 × 56 mm, with hypoechoic fluid and a thinned myometrium (Figure 2). Based on these findings, hematometra was considered the most likely cause of her abdominal pain. Due to complete fibrotic obstruction after radiotherapy, neither mechanical dilatation nor hysteroscopic evaluation was feasible, even with smaller‐caliber instruments. Therefore, ultrasound‐guided uterine puncture was performed under local anesthesia. Approximately 60 mL of blood was aspirated, and the patient's abdominal pain resolved immediately. Although cytology of the aspirated blood was negative, cervical biopsy confirmed recurrent squamous cell carcinoma with pulmonary metastasis. Owing to the coexistence of MDS, chemotherapy was not appropriate, and best supportive care was selected.

Over the following months, hematometra recurred approximately every 2 months, requiring five punctures. Despite the use of local anesthesia, the patient experienced sharp procedural pain, with repeated puncture carrying a risk of infection. Given these limitations, ethanol sclerotherapy was selected as a definitive means of cavity closure.

**FIGURE 3:**
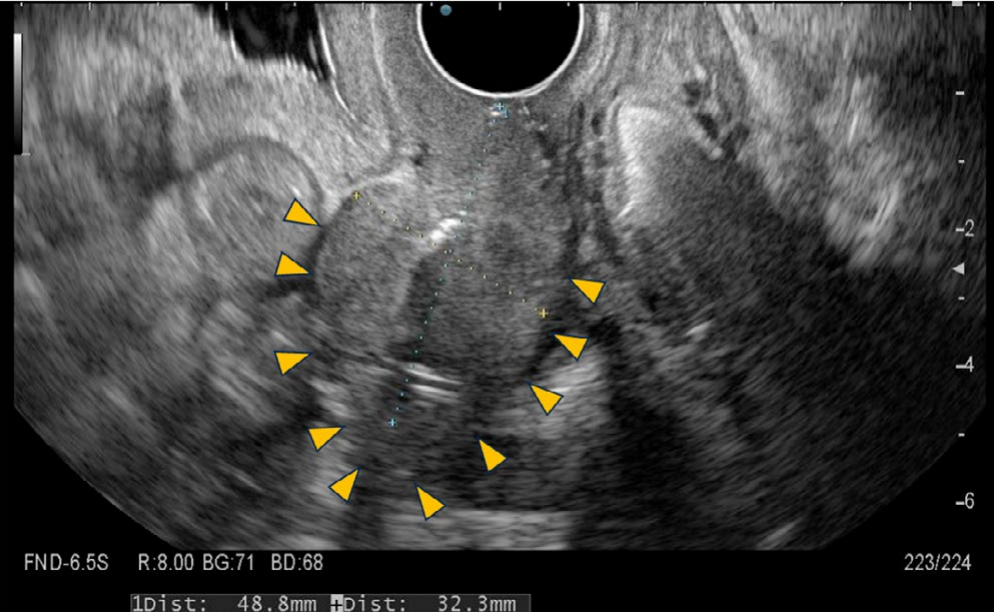
Transvaginal ultrasonography 2 months after ethanol sclerotherapy No echo‐free space was observed in the uterine cavity, and similar findings persisted thereafter. A linear hyperechoic band along the endometrial surface suggests intrauterine adhesion formation that likely prevented recurrence. Yellow arrowheads: Outline of the uterine body.

After the aspiration of 120 mL of intrauterine blood, 12 mL of 100% anhydrous ethanol (Viatris Japan, Tokyo, Japan) was slowly injected under real‐time transvaginal sonographic guidance. The patient remained in the lithotomy position for 15 min, after which approximately 10 mL of ethanol was re‐aspirated. The exposure time was based on a report by Cohen et al. [4], who reported reduced recurrence with retention times ≥ 10 min. No fever, abdominal pain, or other adverse events occurred within 24 h, and she was discharged the following day. Follow‐up ultrasonography demonstrated complete resolution of hematometra with no recurrence for 1 year (Figure 3). Until her death from cerebral hemorrhage secondary to thrombocytopenia related to MDS 17 months after the procedure, hematometra did not recur, and her quality of life remained stable without pain or discomfort from the uterine condition.

### 2.1 Differential Diagnosis, Investigations and Treatment

Differential diagnoses included radiation‐induced cervical stenosis, recurrent squamous cell carcinoma, tumor‐related bleeding, and hematometra associated with MDS. Investigations comprised ultrasonography, computed tomography, cytology, and biopsy. Treatment involved repeated puncture drainage followed by ethanol sclerotherapy when obstruction prevented cervical dilation.

### 2.2 Outcome and Follow‐Up

No recurrence was documented for 1 year after ethanol sclerotherapy, and the patient remained symptom‐free until her death 17 months later.

## 3 Discussion

Although recurrent hematometra requiring definitive surgical management has been reported [4], minimally invasive strategies for preventing recurrence remain poorly described. To our knowledge, this is the first report to demonstrate that ethanol sclerotherapy can achieve sustained resolution in such cases. This approach may represent a practical minimally invasive alternative when repeated drainage is required and cervical dilatation is not feasible.

Hematometra may result from congenital or acquired conditions. In postmenopausal women, cervical stenosis due to uterine malignancy, surgical procedures, or radiotherapy is a common etiology [1]. In this case, cervical enlargement on imaging and squamous cell carcinoma detected on biopsy at hematometra onset indicated recurrent tumor‐related obstruction. Radiation‐induced cervical fibrosis was also likely contributory. Therefore, recurrent tumor, postradiation change, tumor bleeding, and the bleeding tendency associated with MDS were considered possible causes of hematometra with lower abdominal pain.

Owing to the markedly thinned myometrium and risk of severe complications, including infection or uterine rupture in an immunosuppressed state, repeated puncture drainage was initially performed to provide temporary decompression. However, the procedures were painful and carried an ongoing infection risk, prompting the choice of ethanol sclerotherapy as a definitive method of cavity closure.

Ethanol sclerotherapy has been described for multiple cystic or vascular lesions, including ovarian endometriomas [5], renal cysts [6], hepatic cysts [7], thyroid cysts [8], uterine arteriovenous malformations [9], and bleeding gastric ulcers [10] (Table 1). Its mechanism involves cellular dehydration and protein denaturation of epithelial and endothelial cells, which decreases fluid production and promotes thrombotic occlusion of small vessels. In the present case, follow‐up transvaginal ultrasonography revealed a linear hyperechoic band along the endometrial surface (Figure 3), suggesting that intrauterine adhesion formation likely prevented recurrence. Whether ethanol provided any direct therapeutic effect on tumor‐associated vessels remains uncertain.

**TABLE 1:** Review of previously reported studies demonstrating the efficacy of ethanol sclerotherapy.

Site [Reference]	Ethanol concentration (%)	Ratio of injected ethanol volume to cyst fluid volume	The set maximum injection volume
Ovarian endometrioma [4]	95—pure	50%ー80%	100 mL
Renal cyst [5]	99.9	15%	200 mL
Hepatic cyst [6]	95	10%	100 mL
Thyroid cyst [7]	100	33%	10 mL
AVM^a^ [8]	Pure	ー	< 1.0 mL/kg
Gastric hemorrhage [9]	99.5	ー	0.2 mL
Hematometra (present case)	Pure	10%	ー

^a^ AVM, arteriovenous malformation.

The uterus is highly vascular, and its cavity communicates with the peritoneal cavity through the fallopian tubes. This anatomy increases the risk of ethanol entering the systemic circulation or intraperitoneal space compared with that of other organs; therefore, the injection volume must be kept as low as possible, and careful monitoring for systemic absorption or intraperitoneal leakage, which may cause peritonitis, is essential. These risks highlight the need for real‐time imaging guidance and minimal ethanol dosage.

In the present case, a low ethanol dose was chosen based on the lower range of doses reported for other organs (Table 1). A total of 12 mL of 100% anhydrous ethanol, equivalent to 10% of the aspirated volume, was instilled and retained for 10 min, guided by evidence that longer exposure reduces recurrence in ovarian endometriotic cysts [4]. The patient experienced no systemic reaction, peritonitis, or infection, and 1 year of follow‐up confirmed sustained resolution of hematometra.

These findings indicate that ethanol sclerotherapy, when performed with careful procedural control and low ethanol dosage, may offer a safe and effective option for treating hematometra in patients for whom conventional drainage is difficult due to cervical obstruction. Nevertheless, this report only describes a single case, and the optimal ethanol concentration, exposure duration, and monitoring parameters remain unclear. Additional cases and systematic evaluation are needed to clarify the safety profile and therapeutic range of this technique.

## 4 Conclusion

Ethanol sclerotherapy may provide a simple, safe, and minimally invasive option for recurrent hematometra caused by postradiotherapy cervical stenosis and recurrent cervical cancer. Even in patients who are not candidates for invasive procedures, this approach can offer durable symptom relief and help maintain quality of life, underscoring its value as a practical therapeutic alternative.

## Author Contributions

**Fusako Komaru:** data curation; writing – review and editing. **Hirotaka Hamada:** writing – review and editing. **Kojiro Tanabe:** conceptualization; data curation; investigation; writing – original draft. **Hitoshi Niikura:** writing – review and editing.

## Funding

The authors have nothing to report.

## Ethics Statement

In accordance with institutional policy, ethics committee approval is not required for single‐patient case reports.

## Consent

Written informed consent was obtained from the patient's family (the patient was deceased).

## Conflicts of Interest

The authors declare no conflicts of interest.

## Data Availability

Data sharing not applicable to this article as no datasets were generated or analysed during the current study.
